# The Characteristics of Endurance Events with a Variable Pacing Profile—Time to Embrace the Concept of “Intermittent Endurance Events”?

**DOI:** 10.3390/sports12060164

**Published:** 2024-06-13

**Authors:** Joao Henrique Falk Neto, Martin Faulhaber, Michael D. Kennedy

**Affiliations:** 1 Athlete Health Lab., Faculty of Kinesiology, Sport and Recreation, University of Alberta, Edmonton, AB T6G 2R3, Canada; kennedy@ualberta.ca; 2 Department of Sport Science, University of Innsbruck, 6020 Innsbruck, Austria; martin.faulhaber@uibk.ac.at

**Keywords:** surges, sprints, anaerobic power reserve, extreme intensity domain, cycling, triathlon, mountain biking, cross-country skiing

## Abstract

A variable pacing profile is common in different endurance events. In these races, several factors, such as changes in elevation or race dynamics, lead participants to perform numerous surges in intensity. These surges are so frequent that certain events, such as cross-country (XC) skiing, mountain biking (MTB), triathlon, and road cycling, have been termed “intermittent endurance events”. The characteristics of these surges vary depending on the sport: MTB and triathlon require athletes to perform numerous short (<10 s) bouts; XC skiing require periods of short- and moderate-(30 s to 2 min) duration efforts, while road cycling is comprised of a mix of short-, moderate-, and long-duration (>2 min) bouts. These bouts occur at intensities above the maximal metabolic steady state (MMSS), with many efforts performed at intensities above the athletes’ maximal aerobic power or speed (MAP/MAS) (i.e., supramaximal intensities). Given the factors that influence the requirement to perform surges in these events, athletes must be prepared to always engage in a race with a highly stochastic pace. The aim of this review is to characterize the variable pacing profile seen in endurance events and to discuss how the performance of multiple maximal and supramaximal surges in intensity can affect how athletes fatigue during a race and influence training strategies that can lead to success in these races.

## 1. Introduction

The distribution of effort throughout a race is termed pacing, pacing strategy, pacing profile, or pacing pattern [1,2] and is a key factor for optimal endurance exercise performance [3]. When high-level athletes are able to pace themselves in short- (approximately 4 min) to long- (up to 2 h) distance events, the distribution of power output usually follows a J-shaped pattern [4]. The initial section of the race is performed at a higher intensity than the average race pace and represents the fast start [5]. Once this phase is completed, the athletes reduce their intensity and maintain an even pace for most of the race. This phase allows the athletes to recover from the intense effort of the fast start, maintaining an intensity that is sustainable during the race and that allows energy to be conserved for the finishing sprint [5]. This sprint, called the end-spurt, is considered a key race-defining moment [6] where multiple events are won [4,7,8,9].

In many endurance events, however, an even-paced phase does not occur. In these events, the athletes alternate between efforts above and below the average race intensity throughout the race, characterizing a variable pacing profile [1]. These variations in pacing can be so frequent that some endurance events resemble what occurs in team sports [10] and have been referred to as “intermittent endurance events”. The term has been utilized to describe events in cross-country skiing [11], mountain biking [10,12,13], road cycling [14,15], and the cycling leg of different triathlon events [16,17]. Changes in topography, course characteristics, and race dynamics and tactics are some of the factors that ensure that athletes will have to perform several variations in intensity during the race, with the characteristics of these surges unique to each sport.

These surges are performed at intensities that are not sustainable [18], occurring above the maximal metabolic steady state (MMSS) (the intensity associated with the athlete’s critical power (CP) or the 2nd ventilatory threshold (VT2)) or at supramaximal intensities (above the intensity associated with the achievement of maximal oxygen uptake (VO_2_max)) during a graded exercise test, also known as maximal aerobic power (MAP) or speed (MAS). Surges at intensities equivalent to 120 to 160% of the athlete’s MAP are common [10,16,19,20,21], with even higher values (200% to 300% MAP) reported in the literature [10]. A period of low-intensity work (approximately 40% to 60% MAP) [21,22] allows the athletes to recover from the strenuous effort and to cope with the demands of producing frequent bursts of power throughout a race.

Given the frequency, duration, and intensity of these surges, this intermittent profile can have important implications for performance. Compared to performing the same amount of work at a constant intensity, a variable profile leads to greater physiological stress and faster fatigue development and negatively influences subsequent performance [23,24]. A change in the pacing profile might also influence the determinants of performance [3,25], with success in these events related to more than just the traditional factors related to endurance performance (namely, VO_2_max, the intensity associated with the athlete’s lactate threshold (LT) and movement economy) [3]. The ability to perform repeated efforts at a high intensity [8,10,22] and greater anaerobic capacity and power [10,26,27,28] have been hypothesized to be the key to success in these events. The importance of a higher MAP and VO_2_max [11,29,30,31] to performance has also been highlighted. Understanding the specific demands of these races may open new avenues to influence performance in these events [22].

The aim of this review is to characterize the variable pacing profile seen in endurance events and its implications to performance. This review will (1) elucidate the factors that contribute to a variable pacing profile, (2) describe the characteristics (intensity, duration, work-to-rest ratio) of the surges in intensity that occur in these events, and (3) address the consequences of these surges in intensity to endurance exercise performance.

## 2. Methods

This is a narrative review focused on describing the variable pacing profile that occurs in endurance events. A literature review was performed with the following search terms: “variable pacing”, “intermittent pacing”, “pacing pattern”, “pacing strategies”, “power output distribution”, “power profile”, and “power demands”. These terms were combined with “cycling”, “triathlon”, “cross-country skiing”, and “mountain biking”, as events in these sports have been previously described as intermittent endurance events [10,11,13,15,17]. Further, papers on the “physiological demands”, “physical demands”, and “physiological requirements” of these sports were analyzed. Papers were included in the analysis of variable pacing profile if they provided sufficient information to describe the surges in intensity that occur during the races. Subsequently, a manual search within each identified paper was done to find further papers that provided information about the characteristics of the variable pacing profile in these events.

## 3. Factors That Contribute to a Variable Pacing Profile in Endurance Events

Several factors are implicated in the variable pacing profile that is seen in endurance events. While the course’s characteristics provide the most obvious reason for changes in intensity to occur, race dynamics, tactics, and even the influence of governing bodies can contribute to a variable pacing profile.

### 3.1. Out with Old, in with the New—New Race Formats and Changes in Regulations Influenced the Races’ Pacing Profiles

Numerous endurance events have recently been created or modified across different sports to make races more spectacular and spectator friendly [10,17]. Cross-country skiing, for example, had eight out of 12 Olympic events in Sochi 2014 that were different from the 1994 Winter Olympics. Shorter events, such as sprint skiing, and an increase in the number of races with a mass start (10 of the 12 Olympic races now involve mass starts) [32] have increased the demands of surges in intensity and the requirement of sprinting ability in the sport [9,27].

Mountain biking and triathlon have also evolved in their race formats. In Olympic XC MTB (XCO), race duration and lap length were reduced, while the requirement for technical sections in the course increased. Current regulations require races to last between 80 and 100 min, with a lap length of 4–6 km, over a variety of terrains [10,21]. Short track XC MTB (XCC), a new race format introduced in 2018, is performed in loops of no more than 2 km and maximum race times of 20 min [33]. The cycling leg of Olympic and sprint distance triathlons is also performed in shorter loops (3.5 to 5 km) [30], and new race formats, such as super sprints and the team mixed-relay event [34], can be performed in even shorter courses.

The shorter courses have increased the number of tight turns and sharp corners in these events, increasing the number of repetitive, high-intensity accelerations that are performed [10,17,20]. In sprint and Olympics distance triathlon, for example, the number of dangerous curves performed per kilometer has a strong correlation with the variability index (a measure of the variations in power output during a race) and to the number of supramaximal efforts performed [30]. These changes to race formats ensure that several variations in intensity will occur during a race, regardless of the influence of other factors on the races’ pacing profile.

### 3.2. Uphill, Downhill, and Technical Demands—How the Course’s Characteristics Influence Pacing Profile

The technical demands of sports, such as MTB, also contribute to the number of surges that are performed. MTB courses present the athletes with numerous jumps, climbs, descents, and other technical features [13,21]. Navigating these challenges requires the performance of multiple short (8 to 15 s) efforts during the race [12,35]. The fact that the number of surges performed per lap in MTB is not significantly reduced when athletes break into smaller packs corroborates that many of these surges occur as a product of the course’s characteristics [13]. Similar influence of the terrain and technical features have also been reported in cyclocross [36] and off-road triathlon [37].

Further, changes in elevation provide their own challenge in different sports. In cross-country skiing, for example, races must have an equal distribution of flat, uphill, and downhill terrain [32]. The time spent in uphill sections, thus, varies based on the event, with shorter efforts (20 to 40 s) reported in sprint skiing [28,38] and longer efforts (up to 4 min) during longer distance races [39,40]. Likewise, in road cycling, mountainous stages require longer efforts (6 to 10 min) at intensities just above that associated with the maximal metabolic steady state (MMSS), while semi-mountainous stages require shorter (30 s to 2 min), more intense efforts [15].

### 3.3. Breaking Away—The Influence of Race Dynamics to a Variable Pacing Profile

The number and characteristics of the surges might also vary according to the race’s dynamics. Riding in a group leads to a higher number of surges performed as the athletes try to stay within or break away from the pack [14,20]. For example, the four athletes competing as a team in the mixed-relay triathlon performed 17, 11, 8, and 12 surges (>600 W) in intensity during the cycling leg of the race (approximately 11 min) [34]. The athlete who only performed 8 surges was described as chasing a pack, while the others were riding within a group.

The tactics of the chase group might also influence the surges in intensity. In road cycling, it is possible that the group will allow the breakaway to occur earlier in the race, leading to a surge that is less intense [14]. Later in the race, the power output of the surge is higher, and the intensity remains elevated for a further 30 s to 5 min to try to ensure the success of the action [7,14]. As the race nears its end, multiple 5 to 15 s sprints are performed in the 20 min prior to the end-spurt, as the competitors gradually attempt to break away from the pack or position themselves for a successful sprint to the finish line [7,8,41].

Race tactics and dynamics also play an important role in races where position within the packs is important (for example, single-track races where opportunities to pass a competitor are limited), such as MTB [21] and mass-start cross-country skiing [9]. In these events, a longer sprint (around 20 to 30 s) is performed at the beginning of the race as the athletes try to position themselves for the subsequent laps. Athletes might also perform more surges (skiing) or surges that are more intense (MTB) during the initial lap [9,21] to ensure optimal tactical positioning for the remainder of the race. Despite the negative influence that these intense efforts can have on performance, the benefits of competing within the front pack offset the greater metabolic demands of the increased intensity [9,12].

A summary of the factors contributing to surges in intensity and their consequences on the characteristics of the surges is presented in Table 1. A brief analysis of these factors shows that these are intrinsic to the sport (e.g., course characteristics), reflect changes made by governing bodies to make races more spectator friendly, or cannot be predicted (e.g., race dynamics, competitors’ tactics). Even increased media exposure can lead an athlete to attempt a breakaway from the group [14]. As such, athletes must be prepared to engage in a highly stochastic race, with the characteristics of these efforts and their importance for overall performance varying according to the sport and the event.

## 4. Characteristics of Surges in Intensity in Variable Pacing Endurance Events

The characteristics of the surges in intensity that occur during a race vary depending on the sport. The variable pacing profile of events in XC skiing, MTB, road cycling, and the cycling leg of different triathlon races, events referred to as “intermittent endurance events” is described below. An overview of the characteristics of the surges in intensity in these sports is presented in Table 2.

### 4.1. Cross-Country Skiing

Changes in elevation are a key factor in the surges in intensity reported in XC skiing. In sprint skiing races (0.8 to 1.8 km, approximately 3 min in duration) [43], the time spent in individual uphill sections was reported to range between 15 and 50 s, with two different studies [28,38] reporting uphill times of approximately 15, 18, 21, 22, 38, and 51 s. In shorter climbs, intensities of 140–160% of the athletes’ VO_2_max have been reported [28], with only a short period (20 to 40 s) spent in flat and/or downhill sections prior to the next uphill section [28,38]. As the race distance increases, so does the length of the uphill sections. In events ranging from 10 km to 15 km, male and female skiers withstand uphill sections lasting between 40 and 226 s [39,40]. Despite the duration, these efforts are still performed at intensities above the athletes’ VO_2_max but vary based on the length of the section (approximately 115% VO_2_max for longer climbs and 140–160% VO_2_max during shorter ones) [22]. The work-to-rest ratio is similar to that of shorter races. For example, between two uphill sections (42 and 41 s in duration, respectively), athletes competing in a 10 km race spent approximately 25 s in the subsequent downhill section [39]. A short period on a flat or downhill section after a long climb has also been reported in longer races (21.8 km) [9], with a flat and downhill section occurring back to back only twice during the race (14 segments). XC skiing races, thus, are a sequence of uphill–downhill or flat–uphill, with work-to-rest ratios of 2:1, 1:1, and 1:2 reported between climbs [9,28,38,39,40].

In addition to the challenges imposed by the terrain, the increased number of mass start races also influenced the pacing profile in the sport. In these races, narrow tracks often limit the ability to advance in the field. Further, an accordion effect (when competitors in front reduce their speed, but soon accelerate, with the fluctuation in speed propagating backwards) has recently been reported [9]. The accordion effect can lead to additional accelerations and decelerations and also to more incidents during the race for those skiers not in the leading pack [9]. Positional advantage is, therefore, important, and athletes perform the initial lap of the race at an intensity that is higher than that of the subsequent laps, with the higher intensity potentially due to a greater number or intensity of surges performed [9]. Lastly, mass start races are won in the finishing sprint, with several competitors performing an all-out sprint to the finish line. In a 21.8 km race, a group of 10 athletes sprinted over the last 1.2 km of the race, with only 2.4 s separating the top five skiers and a photo finish required to determine the winner [9].

Given its characteristics, success in the sport requires the ability to withstand high intensities (110–160% VO_2_max) during uphill sections and to recovery quickly from these efforts in the downhill sections (40–60% VO_2_max) [11,22,32,44]. These demands are magnified in mass start races where further surges are required to attain a better position in the field. Navigating these demands while retaining the ability to sprint to the finish line is essential for success in these events.

### 4.2. Mountain Biking

The changes to the regulations (reduction in race duration and increase in technical constraints) have altered the demands of the sport. XC MTB races last 80–100 min and are comprised of an explosive start followed by a pattern of intermittent bursts throughout the race [12,21]. The initial burst has been shown to last approximately 68.5 ± 5.5 s, and to occur at an intensity of 481 ± 122 W (6.63 ± 1.34 W/kg, equivalent to approximately 118% of the athletes’ MAP) [12,45]. It must be noted, however, that this data is from prior to the regulation changes. Recent studies have yet to describe the initial surge but have highlighted that the initial lap of the race is performed at a higher intensity than other laps [21,45], due to the performance of numerous surges in intensity that can range between 200 and 300% of the athlete’s MAP, reflecting that athletes need to optimally position themselves early in the race. The benefit of positional advantage for the single-track sections of the race compensates for the negative effects of the intense effort earlier in the race, even if the intensity of some surges approaches the athletes’ maximal anaerobic power (MAnP) [10].

Analyzing the surges in intensity in relation to the athletes’ CP, Naess et al. [13] reported an average of 90 surges in intensity above CP per lap (3.8 km, 16 ± 2 min). These surges had an average duration of approximately 8 s and ranged in intensity from 120% to 140% of the athlete’s CP. Granier et al. [10] described the supramaximal (>MAP) surges over 13 international races (90 ± 9 min) and reported an average of 18 bursts of intensity each lap (laps varied between 3.5 and 5.6 km, with races ranging between 5 and 8 laps). These surges had an average duration of 10 s and were performed at an intensity of approximately 559 W ± 46 W (equivalent to 136% of the athletes’ MAP). Throughout the race, the number of surges performed per lap either remains constant [10] or is reduced [13], while the intensity of the surges is gradually reduced [10,13]. It is not clear if the change in intensity is a result of athletes trying to avoid fatiguing before the end of the race or a result of athletes separating into smaller packs [13].

Combined, these studies indicate that XC MTB requires multiple short (8 to 10 s) efforts above CP and MAP. These occur approximately every 30 to 50 s [13], leading to a work-to-rest ratio of approximately 1:4 or 1:5. Between surges, intensity is reduced to 40% to 60% of the athletes’ MAP, and periods of even lower intensity (no power produced or less than 10% MAP), typically a downhill section, are common [21]. This pattern leads athletes to spend a significant portion of race time (25 to 40%) below their first ventilatory threshold (VT1) or above their MAP (25% to 30% of the race) [10,13,21]. This has led authors to emphasize that the ability to perform numerous surges in intensity above MAP should be a key training goal [10,21]. In addition, the numerous supramaximal surges in the sport have also altered athletes’ profiles, with the MAnP of MTB athletes increasing by 15% over a 10-year period [10,26]. Given the numerous variations in intensity, high-intensity repeatability (i.e., the ability to perform multiple surges in intensity) [46] has been highlighted as the strongest predictor of performance in a group of elite XCO athletes, along with maximal pedaling rate and relative maximal aerobic power [21].

### 4.3. Road Cycling

Road cycling is characterized by prolonged periods of low- and high-intensity exercise and numerous short, high-intensity surges throughout the race [14]. These surges can be as frequent as in other intermittent sports, despite many races in road cycling lasting several hours [8]. The changes in intensity occur when athletes have to overcome varying conditions (uphill sections or headwinds, for example), attempt a breakaway, or have to respond to attacks from other competitors [14]. The characteristics of these surges differ depending on the reason for their occurrence. For example, the variations in intensity that occur due to changes in elevation depend on the race profile. One-day, single-stage, flat races require a higher number of short-duration efforts, with the ability to produce high power outputs in durations ranging from 5 to 30 s being a key factor for performance [15]. Semi-mountainous stages require the ability to perform slightly longer efforts (30 s to 2 min), while time trials and mountainous stages require longer duration (>10 min), sustained, maximal power outputs [15]. The influence of the course’s characteristics is also seen in criterium races, with a greater number of short (6 to 10 s) surges above MAP compared to hilly and flat races (70, 40, and 20 sprints above MAP, respectively) due to the numerous accelerations out of corners in these shorter loop races [47].

The surges in intensity that occur due to race dynamics vary in their characteristics depending on when they occur in the race. Earlier in the race, the surges are performed at a lower intensity since the larger group might allow the breakaway to occur. Later in the race, however, power output remains elevated for a subsequent 30 s to 5 min following an attempted breakaway, to ensure the success of the action [14]. Throughout the race, the characteristics of the surges might also change. In women’s races, the athletes performed 68 efforts of at least 15 s that exceeded 80% of the average power of the final sprint to the finish line, with these being more prevalent during the second (25%), third (26%), and fifth (31%) quintiles of the race [8]. The later stages of the race are particularly challenging. Abbis et al. [14] reported that in the 10 min prior to establishing a breakaway numerous 5–15 s efforts, at a very high intensity (700 to 1000 W, approx. 9.5–14.0 W/kg) are performed. A difference in the amount of time spent at high intensities (>6.6 W/kg) for short efforts (<3.8 s) was also reported between the penultimate and the final 5 min of the race, highlighting the importance of being able to perform multiple short, high-intensity efforts at the end of the race [7,41]. Menaspa et al. [7] also reported that the final 5 min of the race had twice as many surges in intensity when compared to the previous 5 min. The increase in the number of surges in intensity contributes to the last 60 min of the race being, on average, 15% more intense than the other sections of the race [7]. The need to perform multiple short (3 to 10 s efforts) bursts of intensity likely explains why a top-5 or top-10 finish for males and females is largely determined by shorter duration (5 and 10 s) absolute and relative maximal mean power (MMP), even in races with different characteristics (flat vs. mountainous, for example) [48,49].

Particularly in the later moments of the race, team dynamics also play an important role. As a team attempts to win the race with their designated sprinter, the cyclist’s teammates might provide drafting and tactical assistance [50], potentially influencing the demands experienced by the athlete. In the last 60 s preceding the finishing sprint, the position of the cyclist within the bunch (closer to the front of the pack) and the number of teammates in front of the athlete are related to the chances of a successful sprint [51,52]. These last moments of the race include numerous surges in intensity and a higher overall intensity [7,51,52], with the demands likely different between athletes within the same team. It is important to notice that the athlete’s specialization (e.g., climber or sprinter) is another factor to be considered. Compared to climbers and flat specialists, sprinters might possess higher power outputs in short (5 to 30 s) durations, while climbers might be better suited to sustain longer efforts (5 to 60 min) [53,54]. An analysis of the demands of the Tour de France, for example, has shown that sprinters endure a greater load during mountainous stages [55]. In this context, sprinters with good climbing ability might be better positioned to win as other competitors (e.g., flat-terrain sprinters) might be dropped before the finish line [51].

The ability to perform numerous surges in intensity and stay within the leading pack allows the athletes the chance to win the race, by sprinting to the finish line. In women’s races, the average sprint finish required an effort of approximately 20 s in duration, with a peak power output of 886 W (SD 91, range 716–1088, 13.9 W/kg) and an average power output of 679 W (SD 101) or 10.6 (1.5) W/kg [8]. For males, the finishing sprint lasted approximately 13.2 s (ranging between 9 and 17 s), with a peak power output of 1248 W (SD 122) and an average power output of 1020 W (SD 77, range 865–1140) or 14.2 (SD 1.1, 12.2–15.8) W/kg [7,41].

These studies demonstrate that success in road cycling requires several efforts of different durations that vary in their demands according to the race’s characteristics (e.g., flat vs. mountainous) and where they might occur during the race (early in the race vs. in the 5 min preceding the finishing sprint, for example). In addition, a winning performance requires one final sprint to the finish line, performed for an average of 13 to 20 s (for males and females, respectively), and reaching an average intensity that is more than 200% of the athlete’s MAP.

### 4.4. Triathlon

The cycling leg of a super sprint (such as the mixed-relay, MR), sprint (SD), and Olympic (OD) distance triathlons is essential for overall race performance. Positioning at the end of the cycling leg is significantly correlated with race performance [30], and athletes who complete the 2nd transition in the leading pack have a higher probability of winning a medal [56]. This leg is characterized by high variability in power output and cadence, and many short intensity bursts alternated with moderate intensity periods [16,17,20]. However, different methods of classifying these surges lead to large variations in the numbers reported in the literature. Etxebarria et al. [20] found that athletes competing in OD races completed an average of 34 (±14) surges per race, with a surge identified as any 1 s period where the intensity surpassed 600 W (more than 200% of the average race intensity of 252 ± 33 W). The number varied significantly between (ranging from 11 to 55) and within the races, with three athletes in the same event completing 35, 40, and 54 surges. A recent study [30] shows even higher numbers in OD and SD races, with an average of 13.9 peaks of power output above MAP per kilometer. Bernard et al. [16] also reported the duration of surges above the athletes’ MAP, showing that the athletes completed 57 surges of seven seconds and 13 efforts of 15 s throughout the race. In addition, 13 periods of seven consecutive seconds, with intensities above 60% of the athletes’ ManP, were also recorded. Race dynamics also significantly influence the number of surges performed during a race. During the cycling leg of the mixed-relay triathlon (300 m swim, 7 km bike, 2 km run), for example, the athletes perform anywhere between 8 and 17 sprints (depending on their position within the team, which might affect if they are riding with a group or chasing a pack) [34]. The male athletes in the event performed 11 and 12 peaks above 650 W during approximately 10.5 min of the race, while the female athletes performed 8 and 17 bursts of intensity above 400 W, with the difference between the two female athletes due to one athlete being chasing a group while the other was riding with a pack [34]. The fact that athletes perform several efforts even when not racing with a group highlights the influence of the course’s characteristics in the number of surges performed.

The influence of changes in elevation to the variable pacing profile seen in the different triathlon events requires further investigation. Along with a technical course, changes in elevation are responsible for an increased variability in power output in off-road triathlons, with the races resembling what is found in XCO mountain biking [37]. For road events, however, no correlation was found between the athletes’ power profile and the presence of uphill sections [30]. A further factor to be considered is how the athlete’s characteristics and performance on the other legs might influence their race. A strong swimmer might create a gap to the chase pack, leading to a bike leg with little influence from other competitors. The athlete’s locomotor profile [6] might also dictate that some athletes will excel with a variable pacing profile, while others will perform better following a constant pacing effort. Corroborating this assertion, a recent investigation [57] highlighted the importance of determining the order of the athletes within a mixed-team relay to ensure that the athletes that excel in specific circumstances (i.e., racing in a group or in a non-drafting situation) can match the requirements of the race.

The duration of the races and the number of surges reported indicate that the cycling leg of a triathlon event might require athletes to perform a 7 to 15 s effort per minute [16,30,34], with these efforts exceeding the athletes’ MAP [16,34]. In this context, work-to-rest ratios of 1:4 to 1:6 can be expected. Between surges, the intensity is low (approximately 60% MAP) [20,23]. This pattern leads athletes to spend a significant amount of time in intensity zones close to or above their MAP. In the MR and OD races, athletes spent approximately 18% of their race time above MAP [16,20,30], with the amount of work completed in this intensity (as a percentage of total work done in the race) reported to be even higher (37.5%, on average) [30]. Shorter races, such as the MR, might have even higher demands. In the same team, male athletes (positions 2 and 4) were reported to spend 48% and 62% of race time at intensities above 85% of their MAP, while the female athletes (positions 1 and 3) spent 58% and 64% of the race time above this threshold [34].

Further analysis of the surges in intensity during the cycling leg of a triathlon is required as studies have reported peaks of power output above the athletes’ MAP [16,30] or above an arbitrary power output [20,34]. The variations in the characteristics of the surges (duration, frequency, intensity) per lap are also not well established in the literature. For example, the final moments of the cycling leg have been described to occur at a higher intensity than the previous laps, as athletes attempt to position themselves for the start of the running leg [58]. It is possible that this could lead to greater variations in intensity in the final moments of the cycling leg. In turn, this might influence the athlete’s performance during the running leg of the event [17,59].

## 5. Intermittent Endurance Events: Potential Implications for Performance

The completion of numerous surges in intensity has important implications for performance in these events. Two key areas related to performance are highlighted: the development of fatigue during the races and the importance of different determinants of endurance performance to success in these events.

### 5.1. Fatigue Development during the Race

The surges that occur during races are performed at intensities that range from above the MMSS (a surge in intensity at 110% of the athlete’s critical power in XC MTB, for example) [13] to supramaximal intensities, with values up to 300% of the athletes’ MAP reported in the literature [10]. These intensities encompass two different intensity domains: the severe (intensities from the MMSS to approximately 136% of the athlete’s MAP) and the extreme (intensities above 136% of the athlete’s MAP) domains [18,60]. The increased reliance on anaerobic energy production in these domains leads to an accumulation of H^+^, inorganic phosphate (Pi), and blood lactate, along with a drop in phosphocreatine concentrations (PCr) and pH within the muscle [18,61,62]. These factors are implicated in the development of central and peripheral fatigue [61,62].

The magnitude of central and peripheral fatigue is determined by the duration and intensity of the exercise. In self-paced trials, central fatigue is greater following longer duration efforts [63]. Similarly, during repeated supramaximal efforts, peripheral fatigue develops earlier, with central fatigue presenting a later onset [64]. Further, the magnitude of central and peripheral fatigue is dependent on the intensity domain in which the exercise is performed. At task failure, central fatigue is similar following exercise in the moderate, heavy, and severe intensity domains, while it is absent in the extreme intensity domain. Conversely, peripheral fatigue is greater following exercise performed in the severe or extreme intensity domains [61]. Endurance events with an intermittent pacing profile might then present a particular scenario in which the supramaximal surges in intensity will lead to a larger magnitude of peripheral fatigue, while the duration of the exercise (and the continuous demands of subsequent sprints) will also increase the degree of central fatigue. This combination of central and peripheral fatigue is likely to increase the physiological demands of the race and potentially hinder performance.

**Figure 1:**
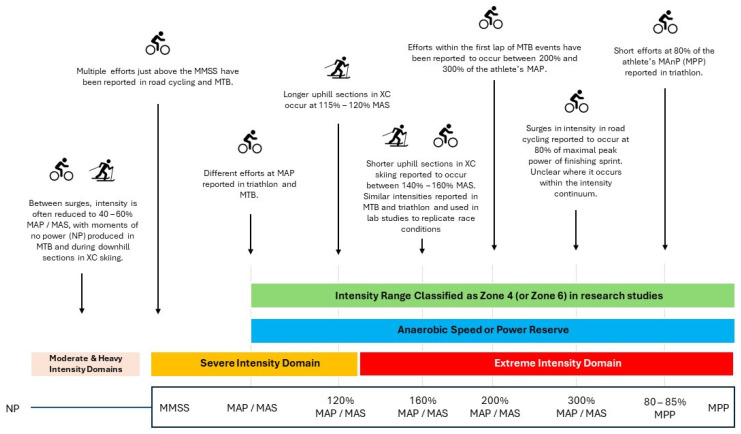
The range of intensities of the surges reported in the literature and their relation to intensity domains and the athletes’ anaerobic power reserve (APR). The image illustrates the numerous surges in the severe and extreme intensity domains and how classifying these as a single intensity zone (green bar) does not accurately represent their physiological demands. NP: no power, MMSS: maximal metabolic steady state, MAP: maximal aerobic power, MAS: maximal aerobic speed, MPP: maximal peak power.

When compared to performing the same amount of work at a constant intensity, a variable pacing consisting of multiple maximal and supramaximal surges leads to higher levels of blood lactate, heart rate, ventilation, oxygen consumption, and perceived exertion [24,59]. Subsequent performance is also impaired to a greater extent [23,24]. Figure 1 provides an overview of reported surges during different events and the intensity they represent.

Further, once exercise intensity exceeds the MMSS, only a limited amount of work, the athletes’ W’, can be performed [18]. The W’ represents a finite work capacity above CP and is related to the accumulation of metabolites related to fatigue, such as inorganic phosphate (Pi), adenosine diphosphate, and hydrogen ions (H^+^) [18,65,66]. A strong relationship between full depletion of W’ and task failure in the severe and extreme intensity domains has been observed [61,65,67]. Importantly, the rate of utilization and the size of W’ might vary based on the intensity of the efforts. The more intense, the faster the depletion, with the W’ for efforts in the extreme intensity domain potentially smaller than for efforts in the severe intensity domain [68]. This anaerobic capacity is attributed to three different energy sources—local oxygen stores (aerobic contribution), high energy phosphates (alactic contribution), and anaerobic glycolysis (lactic contribution) [13,66,69]. During exhaustive exercise, the contribution of these sources is 5–10%, 20–30%, and 60–70%, respectively [11]. While the recovery of local O_2_ stores and PCr is quick (20 s halftime), the recovery of the lactic component of W’ is much slower [65,70]. Efforts that lead to substantial accumulation of blood lactate might also delay the recovery of PCr and impact movement economy [71]. It is best for the athletes, then, that the supramaximal efforts performed are not as intense or prolonged to fully deplete their W’ or to significantly impair their ability to recover from the efforts, hastening their fatigue development.

The level of W’ depletion reported in the literature supports this assertion. In MTB, most of the surges in intensity deplete only a fraction (less than 10%) of the athletes’ W’, and very few efforts reach 50% of their W’ [13]. While the duration of the efforts in the sport remains constant, the intensity is reduced (with a reported W’ depletion of 11% per surge in the initial lap vs. 3–5% throughout the remainder of the race) [13]. A similar pattern occurs in XC skiing, where the magnitude of the depletion of the athlete’s anaerobic capacity (assessed through the maximal accumulated oxygen debt (MAOD)) in each surge is considered small (approximately 50% or less) in relation to the athlete’s total anaerobic capacity [11,22]. The accumulated depletion of anaerobic sources during a race, however, is much greater than the athletes’ capacity. Athletes can expend up to 3.8 times their anaerobic capacity during a XC skiing race [11], with similar levels of expenditure and replenishment of the W’ also reported in MTB [13] and off-road triathlon races [37].

**Figure 2:**
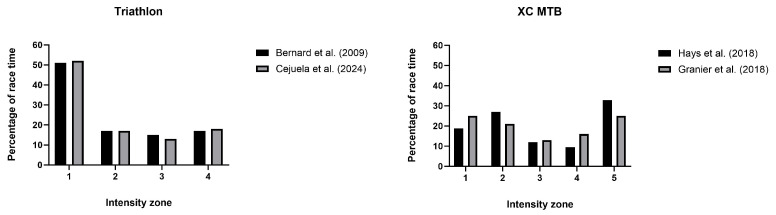
Time spent at different intensity zones during intermittent endurance events in triathlon (top) and mountain biking (bottom). Data for triathlon from Bernard et al. [16] and Cejuela et al. [30]; data for mountain biking from Granier et al. [10] and Hays et al. [21]. Training zones in triathlon correspond to: Z1: below VT1, Z2: between VT1 and VT2, Z3: between VT2 and MAP, Z4: above MAP. In MTB: Z1: no power or 10% below MAP, Z2: 10% MAP to below VT1, Z3: VT1 to VT2, Z4: VT2 to MAP, Z5: above MAP.

This greater level of anaerobic capacity expenditure is possible because the intense efforts are interspersed with periods at low intensity. This pattern of intense surges and low-intensity efforts leads athletes to spend a significant amount of race time at intensities above the MMSS and their MAP/MAS, along with long periods at intensities below the first ventilatory threshold (in the moderate-intensity domain) (Figure 2). At these lower intensities, athletes can recover their W’ and minimize the increases in muscle activity and oxygen consumption [65,66]. W’ reconstitution occurs in a biexponential way, with a faster initial recovery (e.g., a 30% reconstitution in the first 30 s after exercise to exhaustion), followed by a slower recovery of the remaining portion [70]. Recovery of fatigue-related substrates (e.g., resynthesis of PCr) and clearance of fatigue-related metabolites from muscle (e.g., H^+^) also occur [65,66,69,70], allowing the athletes to avoid the attainment of a limiting intramuscular environment. The reduction in the intensity of the surges throughout the races has been hypothesized to occur so that athletes avoid achieving this level of metabolic stress [13]. In this context, the ability to minimize the metabolic disturbances due to repeated efforts above the MMSS and at supramaximal intensities is essential to performance in these events.

### 5.2. Impact of a Variable Pacing Profile on Determinants of Endurance Performance

**Figure 3:**
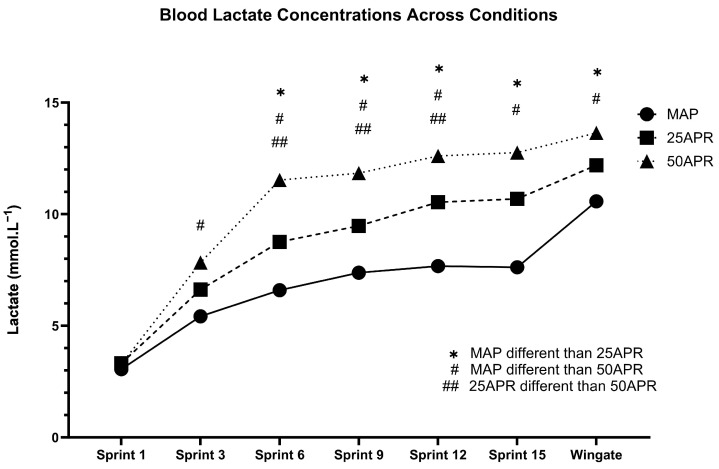
Blood lactate concentrations during a repeated sprint protocol performed, consisting of fifteen 10 s sprints at three different intensities. MAP: maximal aerobic power, 25APR: intensity associated with 25% of the participant’s APR; 50APR: intensity associated with 25% of the participant’s APR.

**Figure 4:**
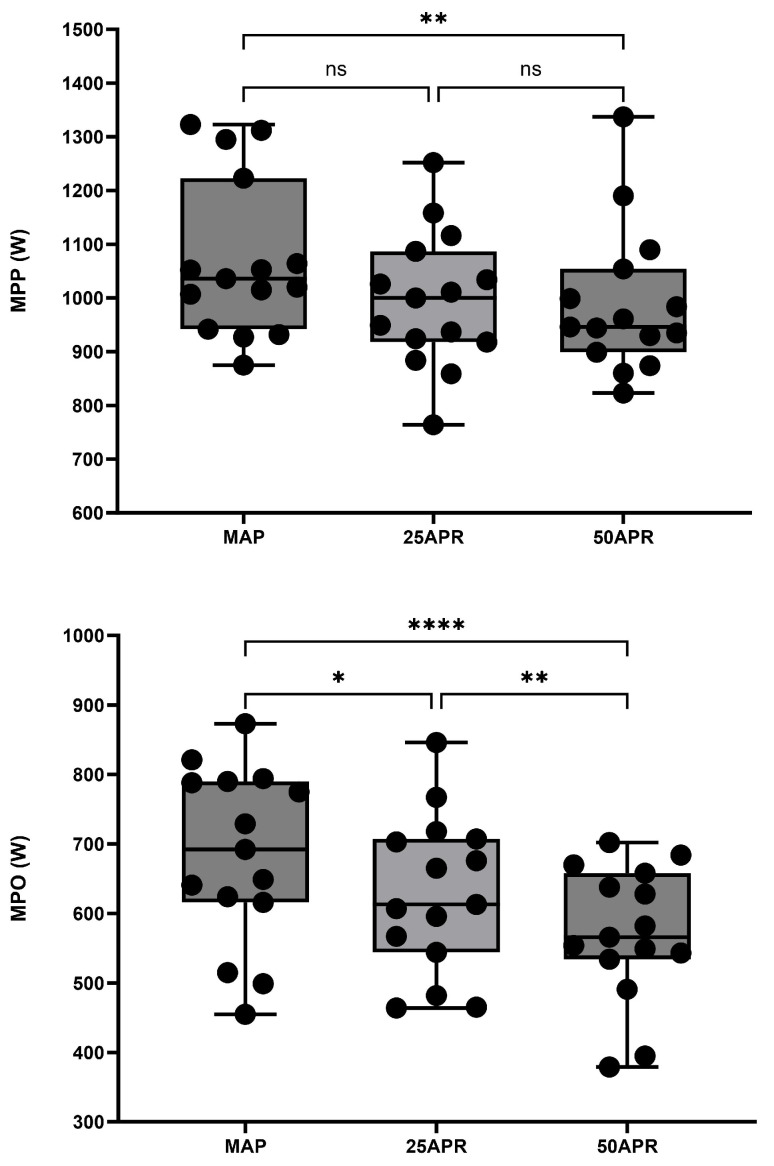
Performance during a 30 s Wingate, following the completion of the repeated sprint protocol. Each circle represents a data point. (**Top panel**) maximal peak power (MPP); (**bottom panel**) mean power during the 30 s effort. * *p* < 0.05, ** *p* < 0.01, **** *p* < 0.001, ns = non significant.

A change in the distribution of power output during an endurance event alters the contribution of different factors that determine performance [3,25]. In endurance events with a variable pacing profile, the athletes’ MAP/MAS and VO_2_max might have an increased importance to performance. A higher MAP and VO_2_max improves recovery following repeated sprints [72,73,74] and contributes to faster recovery of W’ [69,70], while also allowing athletes to minimize the number of supramaximal efforts they complete. As time to fatigue in supramaximal efforts is related to the percentage of the APR at which the efforts are completed [6,75], a higher MAP might lead to efforts performed at a lower % of the athlete’s APR. Unpublished data from our lab show that performing sprints at a higher supramaximal intensity leads to greater physiological stress and hinders subsequent performance. In the study, 15 well-trained (tier 2) [76] male endurance athletes completed a protocol simulating the work-to-rest ratio reported in some intermittent endurance events [10,16]. The participants were asked to complete fifteen 10 s sprints, interspersed with 50 s of low-intensity cycling (60% MAP). The protocol was performed under three different conditions, depending on the intensity of the sprints, either at the intensity associated with the athletes’ MAP or at 25% or 50% of their APR. Blood lactate concentrations showed a significant difference between conditions (Figure 3). Efforts at MAP were well-tolerated by all participants, but supramaximal intensities showed significantly higher blood lactate levels. Subsequent performance in a 30 s all-out effort was also significantly impaired following supramaximal efforts (Figure 4). Current recommendations to training in intermittent endurance sports corroborate these results. For MTB athletes, the performance of high-intensity interval training to enhance MAP has been recently emphasized [10,21], and similar recommendations have been made in triathlon [30] and XC skiing, where a higher VO_2_max is also highlighted [28,77].

Further, the increased reliance on anaerobic energy is reflected in the changes to performance determinants over the years. Anaerobic power (MAnP) and maximal velocity (V_max_) have been identified as important determinants of performance in MTB and XC skiing [19,29], respectively. For MTB athletes, MAnP has increased by 15% over a 10-year period [10,13]. Increasing an athlete’s MAnP also raises the upper boundary of the APR, potentially leading athletes to perform supramaximal efforts at a lower percentage of their APR. The performance of intense efforts relying on anaerobic energy also increased the importance of anaerobic capacity to performance in these two sports [26,27,28,32].

The ability to perform repeated efforts at intensities above CP and MAP is also essential to performance in these events. The development of repeated sprint ability (RSA) can benefit athletes involved in road cycling [8,14], MTB [10,21], XC skiing [11,19,22], and triathlon [17,30]. RSA training could improve the factors that limit the performance of multiple surges during a race (e.g., oxidative capacity, H^+^ buffering) and neural factors such as muscle activation and recruitment strategies [78]. Different strategies such as short- and long-duration high-intensity interval training [79,80] and resistance training [80,81] can be utilized to improve these factors.

Lastly, the ability to tolerate the intense demands of the race without a significant decrement in performance is key to success. This trait, called physiological resilience [82] or durability [46], has been proposed as the fourth determinant of endurance performance [82]. This resiliency is related to the degree of change or decoupling in physiological responses (e.g., heart rate, blood lactate) to the same exercise intensity as work is accumulated during a race [46]. To date, only a few studies have highlighted how these responses change during endurance exercise, with results showing a shift in the athlete’s physiological responses at the first [83] and second [59] thresholds as work is accumulated. When compared to exercise performed at a constant intensity, Etxebarria et al. [59] demonstrated that the magnitude of the shift in MMSS is greater after a variable intensity exercise bout with multiple supramaximal surges in performance, even when both protocols are matched for total workload. A recent study with road cyclists showed similar results, with greater decrements in high-intensity performance following variable work [84]. Indeed, it has been hypothesized that while the absolute intensity of the surges is reduced throughout a race in MTB [13], it is possible that the efforts still represent the same relative intensity, as the intensity associated with the athlete’s MMSS might have changed due to the work already performed. The ability to perform and recover from numerous surges in intensity and its relationship with durability and performance warrants further investigation.

## 6. Practical Applications and Future Directions

### 6.1. Summary of Different Intensity Zones to Performance

A variable pacing profile is common in many endurance events. In these races, multiple surges in intensity are performed, with the characteristics (intensity, duration, frequency, work-to-rest ratio) of these surges varying depending on several factors (e.g., race dynamics, course profile). As such, athletes must be prepared to always engage in a highly stochastic race. These surges can occur so often that the pacing profile of certain events resembles what is seem in team sports, leading to these sport being characterized as “intermittent endurance events” [10,11].

The physiological demands of these intermittent endurance events, however, are not yet fully understood. Of the studies that compared the responses between constant and variable intensity endurance exercise, only a few [23,24,59] utilized protocols that replicate the variations in intensity seen in these events. While challenging [14], future studies should aim to replicate the duration, intensity, and work-to-rest ratio of these races. Further, the performance of supramaximal efforts is common in these events. These efforts occur within the athletes’ APR. This range of intensities (from MAP to MPP) encompasses two different exercise domains (the severe and the extreme intensity domains). The factors that limit exercise within these domains are distinct in nature, and as such so are the fatigue mechanisms associated with them [61]. Distinct intensity zones might even exist within the extreme intensity domain [85]. In addition, the intensity that demarcates the severe and the extreme intensity domain is not clear, with recent studies showing that this threshold can occur at a lower percentage (120 to 130%) of the athlete’s MAP [61,85] than previously thought (approximately 136% MAP) [60]. Nevertheless, current studies report the amount of time spent at supramaximal intensities as a single intensity zone (zone 4 or zone 6, in either a 3- or 5-zone model, respectively) (Figure 1) [10,13,16,21,30]. The intensity of these efforts has also been arbitrarily classified in different studies. For example, Peiffer et al. [8] classified some of the supramaximal efforts in road cycling based on a percentage of the power output of the finishing sprint in the race. In triathlon, intense efforts have been described as surpassing a certain threshold (400 W or 600 W) [20,34] or as a percentage of the athletes’ MAnP [16]. Classifying these efforts according to a percentage of the athletes’ APR might provide a method to standardize the intensity of the efforts, while elucidating the expected physiological demands of these surges [6].

### 6.2. Potential Aids for Performance Improvement and Environmental Considerations

Understanding the demands of intermittent endurance events might open new avenues for interventions that can influence performance. For example, creatine supplementation, long considered to not have an important effect on endurance performance, can enhance performance in events where multiple surges in intensity are performed [86,87]. Given their intermittent profile, training interventions that can improve performance in team sports, such as repeated sprints with blood flow restriction or in hypoxia [88,89], can also become valuable tools to enhance performance in intermittent endurance events. Lastly, heat and altitude have both been demonstrated to reduce performance during repeated sprint efforts [90,91]. Further research should investigate the influence of these environmental factors during intermittent endurance events, with particular focus on how they might influence the ability to perform multiple surges in intensity and their effect on subsequent performance.

### 6.3. Limitations

This review aimed to describe the variable pacing profile that occurs in different endurance events, with a particular focus on events described as “intermittent endurance events”. Some limitations within the literature must be acknowledged. First, the research in this topic has been done with athletes that ranged from trained to elite or world class [76]. It is unclear if races performed at lower levels of the sport present a similar pacing profile. As such, caution is advised when translating this information to recreational- or developmental-level athletes. Second, it is important to consider the limitations of a narrative review in relation to methodological rigor and the selection of articles for inclusion. Nevertheless, we are confident that our review addresses best practices in writing a narrative review [92] and that it provides a key contribution to deepening the understanding of this topic [93]. Future studies in each individual sport presented here (and others in the literature) are necessary for a better understanding of the specific demands of endurance events with a variable pacing profile.

## Figures and Tables

**Table 1:** Summary of factors that contribute to a variable pacing pattern in intermittent endurance events and how it affects the characteristics of the surges.

Factors Contributing to Surges	Effect on Variable Pacing Pattern	Influence on Characteristics of Surges	Sports Influenced by It
Changes in elevation/topography	Variations in intensity according to the duration and length of the climb	Performance of short- (<15 s) (MTB), moderate- (30 s to 2 min), and long- (>2 min) (XC skiing, road cycling) efforts during the race	MTB, XC skiing, Road cycling
Course’s characteristics	Repetitive accelerations, tight turns, dangerous curves, technical sections	Performance of multiple short (<15 s) efforts	Triathlon, MTB
Race format	Mass start races, competing in shorter loops	Performance of multiple short (<15 s) efforts, end-spurt determines winner	MTB, XC skiing, Road cycling
Race tactics/dynamics	Tactical positioning, breakaways, pack riding	Longer and more intense surges in first lap (tactical positioning), less intense and shorter surges earlier in the race (breakaway), higher number of surges prior to finishing sprint, need to sustain higher intensity following surge later in the race	MTB, XC skiing, Road Cycling, Triathlon

**Table 2:** Characteristics of variable pacing profile in different sports.

Study	Participants and Competition Level	Race Characteristics		Characteristics of Surges				Time Spent/Work Done in Each Intensity Zone
		Distance/Average Duration	Average Intensity	Number	Duration	Intensity	Recovery Duration/Work to Rest Ratio	
Triathlon								
Mixed Relay (MR)								
Sharma & Périard [34]	4 elite (2 males, 2 females) World Championships	Males: 10.5 min Females: 11.5 min	NR	11 and 12 (males) 17 and 8 (females)	NR	>650 W >400 W (8 W/kg)	NR	48% and 62% above 85% 4MMAP (males) 58% and 64% above 85% 4MMAP (females)
Sprint (SD) and Olympic Distance (OD)								
Bernard et al. [16]	10 Elite triathletes (5 males, 5 females) World Cup	40 km 72 min females 63 min males	66.0 ± 7.1% MAP L1-L2 60.7 ± 9.1% MAP L3-L4 52.7 ± 7.5% MAP L5-L6	44 13 13	7 s 15 s 7 s	>100% MAP >100% MAP >60% MAnP		Z1: 51 ± 9% Z2: 17 ± 6% Z3: 15 ± 3% Z4: 17 ± 6%
Etxebarria et al. [20]	5 elite male triathletes (12 race profiles from 7 ITU international races)	40 km	252 ± 33 W (3.9 ± 0.5 W/kg)	34 ± 14 *	NR	>600 W	NR	NR
Cejuela et al. [30]	4 male triathletes 13 WTS races (6 SD, 8OD) Tokyo 2021 Olympic Games (OD)	Approx. 40 km (average of 8.86 laps per race) for OD Approx. 20 km (average of 5.4 laps per race) for SD	58.3% MAP (mean power) 65% MAP (normalized power) Athlete’s mean MAP across study: 450 W	Average of 13.9 ± 3.6 peaks (surges) per km	NR	Peaks reported as efforts above MAP Power profile during races— 5 s MMP: 795 ± 102 W (approx. 176% MAP) 30 s MMP: 499 ± 62 W (approx. 110% MAP) 60 s MMP: 411 ± 48 W (approx. 91% MAP)	NR	Time Z1: 51.9 ± 6.5% Z2: 17.3 ± 3.9% Z3: 13.3 ± 2.6% Z4: 17.4 ± 5.0 Work done Z1: 22.0 ± 5.8% Z2: 20.4 ± 4.0% Z3: 20.0 ± 3.5% Z4: 37.5 ± 10%
Smith et al. [42]	3 elite triathletes (1 male, 2 females) ITU World Cup	40 km (6 laps)	Male: 238.3 ± 167.4 W Female 1: 229 ± 111 W Female 2: 225 ± 124 W	Male: 8 (a single lap) Females: Numerous per lap (NR)	NR	Male: 600 W (threshold power estimated to be 320–350 W) Females: >500 W		NR
Mountain Biking								
Granier et al. [10]	8 male (5 U23, 3 elite) 13 international races	5 to 8 laps 28.15 ± 5.41 km 90 ± 9 min	283 ± 22 W 68 ± 5% MAP	18 ± 4 (per lap)	10 s ^$^	559 ± 46 W	Every 40 ± 14 s	Z1: 25 ±5% Z2: 21 ± 4% Z3: 13 ± 3% Z4: 16 ±3% Z5: 26 ± 5%
Næss et al. [13]	5 male, 2 females (23.4 years, 68.5 kg), National Standard	3.8 km Loop 19 km for females (5 laps) 23 km for males (6 laps) 96 ± 7 min (lap time: 16 ± 2 min)	249 ± 63 W 3.6 ± 0.7 W 180 ± 4 bpm 63 ± 4% MAP 76 ± 9% CP	Approx. 90 per lap (above CP, not supramaximal) Starting loop had 17 ± 3	8 s (5.2–11.6)	1.18 to 1.41 (Fraction of CP) SL: 1.41 ± 0.07 Remainder of race has an average of 1.2	NR	Zero PO: 27% ± 3% Time > CP: 40 ± 8% Time > MAP: 26 ± 8%
Hays et al. [21]	16 male juniors or U23 (national or international level) Simulated race in official racetrack	3 laps (5.1 km) Simulated: 64 ± 1.5 min Competition: 66 ± 2 min	NR	22.1 11.8 5.7 3.1 1.8	1–5 s 5–10 s 11–15 s 16–20 s >20 s	>MAP (specific intensity not reported)	NR	L1, L2, L3, respectively: NP: 18.8 ± 4.3%, 18.9 ± 4.6%, 19.8 ± 6.0% Z1: 27.0 ± 8.1%, 31.2 ± 9.8%, 33.5 ± 10.2% Z2: 11.9 ± 4.9%, 12.3 ± 5.2%, 13.6 ± 5.0% Z3: 9.5 ± 5.1%, 9.7 ± 4.3%, 9.1 ± 4.5% Z4: 32.8 ± 8.2%, 27.9 ± 7.9%, 24.0 ± 8.2%
Road Cycling								
Peiffer et al. [8]	7 professional female cyclists	31 races where the rider of interest finished in the top-5. Average race time of 179.4 ± 33.4 min	167 ± 24 W	68 efforts above 80% of the maximal final sprint (numerous 5-, 15-, 30-, 60-, 240-, and 600-second efforts above 80% MMP80 also occurred)	15 s	>80% of MSP 80 (80% of the PO of the final sprint) Other sprints were above 80% of mean maximal power for specific duration.	NR	NR
XC Skiing ^#^								
Sprint Skiing								
Sandbaak et al. [28]	12 elite male XC skiers (3 WCs)	240 ± 5 s (234–248); 1820 m.		3 (S3, S4, S7)	S3: 18.8 ± 0.5 s S4: 51.4 ± 2.3 s S7: 15.0 ± 0.7 s Total of 85.2 ± 3.1 s	160% VO_2_max (476 ± 42 W) for S4 Not reported for S3 and S7.	NR (S5 + S6, approx. 50 s–1:1)	NR 36% Uphill (>MAP) 27% Flat 30% Downhill 7% Curved
Ihalainen et al. [38]	11 female XC skiers (Scandinavian Cup)	250.4 ± 5.8 s	NR	3 (S2, S5, S7)	S3: 21.1 ± 0.9 s S5: 22.1 ± 0.9 s S7: 38.2 ± 2.0 s	NR	NR S3 + S4: approx. 41.5 s S6: 14.1 s	NR
Distance Skiing								
Sandbaak et al. [39]	10 elite females (highest ranked in the world to top-15 Norway) VO_2_max = 68.0 ± 4.8 mL/kg/min	10 km (2 × 5-km laps) Total of 56% uphill (483 ± 31 s), 16% flat (193 + 10 s), 28% downhill (218 ± 8 s) Total of 894 s	NR	5 (per lap)	S3: 42 ± 2 s S5: 41 ± 3 s S7: 162 ± 10 s S9: 152 ± 11 s S14: 85 ± 4 s Total of 483 ± 31 s	NR	S4 = 25 s (downhill) S6: 46 s (downhill) S8: 31 s (downhill) S10–S13: 128 s (downhill and flat)	NR 56% uphill (>CP/MAP) 16% flat 28% downhill
Staunton et al. [40]	19 (9 female, 10 male) tier 3 athletes, FIS-sanctioned	Approx. 4900 m per lap Men: 3 laps (14,678 m) Women: 2 laps (9743 m) Total of 165 m of climbing Total Time Women: 28 min 44 ± 58 s Men: 38 min 37 ± 57 s	NR	4 (per lap) S1, S3, S5, S7	Women S1: 226 ± 10 s S3: 67 ± 3 s S5: 243 ± 11 s S7: 61 ± 3 s Men S1: 194 ± 5 s S3: 56 ± 2 s S5: 210 ± 10 s S7: 52 ± 2 s	NR	Women S2: 102 ± 2 s S4: 36 ± 1 s S6: 55 ± 1 s S8 + S9: 86 s Men S2: 94 ± 3 S4: 33 ± 1 s S6: 51 ± 1 S8 + S9: 78 s	NR

4MMAP: 4-min maximal aerobic power (surrogate of maximal aerobic power(MAP)); NR: not reported; SL: starting lap; L1—LN: lap number; CP: critical power; S1—SN: section number; MMP: maximal mean power over different durations; MSP: maximal sprinting power of the finishing sprint; Z1 to Z5: intensity zones; ^#^: surges reported in XC skiing represent duration of uphill sections in different events; *: average; ±: standard deviation of the races reported in the study; ^$^: value reported via personal communication with the author.
